# Causal relationship between gut microbiota and gynecological tumor: a two-sample Mendelian randomization study

**DOI:** 10.3389/fmicb.2024.1417904

**Published:** 2024-08-07

**Authors:** Yajun Xiong, Xiaonan Zhang, Xiaoya Niu, Long Zhang, Yanbing Sheng, Aiguo Xu

**Affiliations:** Department of Respiratory and Critical Care Medicine, The First Affiliated Hospital of Zhengzhou University, Zhengzhou, China

**Keywords:** Mendelian randomization, gut microbiota, gynecological tumor, uterine fibroids, ovarian cancer, *Lachnospiraceae*

## Abstract

**Introduction:**

Previous research has established associations between alterations in gut microbiota composition and various gynecologic tumors. However, establishing a causal relationship between gut microbiota and these tumors remains necessary. This study employs a two-sample Mendelian randomization (MR) approach to investigate causality, aiming to identify pathogenic bacterial communities potentially involved in gynecologic tumor development.

**Methods:**

Data from the MiBioGen consortium’s Genome-Wide Association Study (GWAS) on gut microbiota were used as the exposure variable. Four common gynecologic neoplasms, including uterine fibroids (UF), endometrial cancer (EC), ovarian cancer (OC), and cervical cancer (CC), were selected as outcome variables. Single-nucleotide polymorphisms (SNPs) significantly associated with gut microbiota were chosen as instrumental variables (IVs). The inverse variance-weighted (IVW) method was used as the primary MR analysis to assess the causal relationship. External validation An was conducted using an independent. Sensitivity analyses were performed to ensure robustness. Reverse MR analysis was also conducted to assess potential reverse causation.

**Results:**

Combining discovery and validation cohorts, we found that higher relative abundance of Lachnospiraceae is associated with lower UF risk (OR: 0.882, 95% CI: 0.793-0.982, P = 0.022). Conversely, higher OC incidence is associated with increased relative abundance of Lachnospiraceae (OR: 1.329, 95% CI: 1.019–1.732, P = 0.036). Sensitivity analyses confirmed these findings’ reliability. Reverse MR analysis showed no evidence of reverse causation between UF, OC, and Lachnospiraceae.

**Discussion:**

This study establishes a causal relationship between Lachnospiraceae relative abundance and both UF and OC. These findings provide new insights into the potential role of gut microbiota in mechanisms underlying gynecological tumors development.

## Introduction

In recent years, the incidence and mortality rates of gynecological tumors have been on the rise, posing a significant threat to women’s health. Among these tumors, uterine fibroids (UF) are the most common. Cervical cancer (CC), endometrial cancer (EC), and ovarian cancer (OC) are the three most prevalent gynecologic malignancies and rank among the top 10 in female cancer incidence (Chen et al., 2016). The occurrence of gynecological tumors is influenced by various factors, including genetics, hormonal imbalances, obesity, persistent HPV infection, and chronic conditions such as endometriosis.

The human microbiome resides in specific body areas and is essential for nutrient absorption, preserving epithelial integrity, detoxification, regulating inflammation and immunity, and defending against pathogens (Baker et al., 2017; Thursby and Juge, 2017). With advances in next-generation sequencing (NGS) technologies, we have gained a deeper understanding of the human microbiome, particularly the gut microbiome, which is dominated by *Bacteroidetes* and *Firmicutes* (Russell et al., 2013). A healthy gut microbiota produces short-chain fatty acids (SCFAs) through fermentation, maintaining gut acidity, promoting beneficial bacteria growth, inhibiting pathogen colonization, and promoting epithelial regeneration. In summary, a healthy gut microbiome significantly suppresses chronic inflammation, obesity, metabolic syndrome, and cancer-related diseases (Rinninella et al., 2019).

Microbial communities typically maintain a symbiotic balance within the host. However, several factors can impact the microbiome composition, including medication, obesity, diet, exercise, and genetics (Rinninella et al., 2019). Intestinal dysbiosis, characterized by reduced diversity and stability of gut microbiota, can lead to the overgrowth of harmful bacteria and the production of specific by-products, resulting in immunological and metabolic disturbances. It has been linked to inflammatory bowel disease, diabetes, obesity, metabolic syndrome, and cancer (Gilbert et al., 2018). Recent research has emphasized the connection between gut microbiota and tumors. Previous studies have confirmed the role of microbial dysbiosis in gastrointestinal tumors such as colorectal and liver cancer, as well as tumors outside the digestive tract, including skin, mouth, lung, and reproductive cancers (Sobhani et al., 2019). The influence of microbes on cancer mechanisms is intricate. In gynecological tumors, factors such as modulation of inflammatory responses, DNA damage, impacts on estrogen levels, and production of toxins and metabolites can disrupt the equilibrium of gut microbiota (Borella et al., 2021).

The gut microbiome influences gynecological tumor development. Comparing UF to healthy individuals, we found reduced *Bifidobacteria scardovii*, *Ligilactobacillus saerimneri*, and *Lactococcus raffinolactis*, and increased *Pseudomonas stutzeri* and *Prevotella amnii* (Mao et al., 2022). Sims et al. (2019)’s study revealed more *Prevotella*, *Porphyromonas*, and *Dialister*, and decreased *Bacteroides*, *Alistipes*, and *Lachnospiracea* in CC. Zhao identified gut microbiota differences in EC patients, with enriched *Ruminococcus* as a prognostic biomarkers (Zhao et al., 2022). *Proteobacteria* and *Veillonella* were more common in cachexia among breast and ovarian cancer patients (Ubachs et al., 2021). Chambers et al. (2022)’s study demonstrated gut microbiota dysbiosis in OC leading to tumor progression and cisplatin resistance.

However, these findings are primarily from cross-sectional studies and cannot establish causation. Determining causality is crucial for comprehending gynecological tumor development and guiding microbial interventions. We employed Mendelian randomization (MR) analysis to elucidate the causal relationship between gut microbiota and gynecological tumors, utilizing genetic variation as instrumental variables (IVs) to avoid confounding and reverse causality (Chen et al., 2020). This study aims to clarify the connection between gut microbiota and gynecological tumors, providing a theoretical foundation for potential therapeutic strategies.

## Materials and methods

### Study design

In this study, we explored the causal relationship between gut microbiota and four common gynecological tumors (UF, EC, OC, and CC) using MR analysis. The gut microbiota was the exposure, SNPs were significantly associated with the exposure, and UF, EC, OC, and CC were the outcomes. We examined both the discovery and validation cohorts of gynecological tumors. The genetic IVs needed to satisfy specific criteria: (1) being significantly associated with the gut microbiome; (2) being independent of all confounding factors except the gut microbiome; and (3) influencing outcomes only through the gut microbiome. Additionally, we performed reverse MR analysis to investigate the causal relationship between these tumors and gut microbiota.

### Data sources

Genetic summary data for gut microbiota were obtained from MiBioGen,^1^ a large scale, multiethnic GWAS meta-analysis. The study included 18,340 participants from 24 cohorts, with approximately 78% from Europe. We analyzed microbial composition in three different variable regions of the 16S rRNA gene, encompassing 211 taxonomic groups, including 9 phyla, 16 classes, 20 orders, 36 families, and 131 genera. Adjustments were made for age, sex, technical covariates, and genetic principal components.

Genetic summary data for the UF, EC, OC, and CC discovery and validation cohorts were sourced from the IEU Open GWAS Project.^2^ The data used in this study are publicly available GWAS summary data and have received ethical approval.

### Selection of instrumental variables

To ensure the authenticity and accuracy of conclusions regarding the causal relationship between the gut microbiome and the risk of gynecological tumors, we employed quality control measures to select optimal IVs: (1) SNPs below the genome-wide statistical significance threshold (5 × 10^–8^) were chosen as IVs, resulting in a limited number of gut microbiota selections. To comprehensively explore the connections between gut microbiota and tumors, we applied a secondary threshold, selecting SNPs below the locus-wide significance level (1 × 10^–5^) to uncover additional potential causal associations (Sanna et al., 2019). (2) To mitigate bias from linkage disequilibrium (LD) among IVs, we set the LD coefficient to r^2^ < 0.1 and the region width to 500kb, ensuring independence between SNPs and preventing pleiotropy (Hemani et al., 2018). (3) SNPs were excluded if their *P* value for the outcome was less than 0.05 (Hartwig et al., 2016). (4) SNPs with inconsistent alleles between exposure and outcome samples (i.e., A/G vs. A/C) were excluded. (5) Palindromic A/T or G/C alleles were also excluded. (6) To assess the strength of the selected SNPs, we calculated the *F* statistics for each bacterial taxon using the formula:


$$F\,\frac{R\,2\,\left(n-1-k\right)}{\left(1-R\,2\right)\,k}$$

where *R*^2^ is the portion of exposure variance explained by the IVs, n is the sample size, and k is the number of IVs. An *F* statistic ≥ 10 indicates no strong evidence of weak instrument bias. IVs with *F* statistics <10 were considered weak and excluded (Burgess and Thompson, 2011).

### MR analysis

To investigate causal relationships between the gut microbiome and UF, EC, OC, and CC, we used three regression models: IVW, Weighted Median Estimation (WME), and MR-Egger regression, respectively. The IVW method was the main approach, with the other two methods serving as complementary approaches. The IVW method used the inverse variance of each IV as weights to calculate the summary causal effect estimate. The WME method applied weighted median estimation, requiring at least 50% of valid IVs and ordering SNPs based on their weights before selecting the median as the result (Bowden et al., 2016). The MR-Egger regression estimated the general linear regression model by calculating the correlation coefficients between each SNP and the outcome, as well as between the SNP and the exposure.

We validated the significant bacterial genera identified in the discovery cohort and used the same MR analysis methods in the validation cohort to ensure consistency and reliability of the findings.

### Pleiotropy, heterogeneity and sensitivity analysis

We conducted sensitivity analyses to assess the robustness of our results. Firstly, we used Cochran’s Q test to assess SNP heterogeneity, considering a *P* value below 0.05 indicative of heterogeneity (Bowden et al., 2017). Secondly, we employed MR-Egger regression and MR-PRESSO to detect and address potential horizontal pleiotropy. MR-Egger regression assesses whether genetic instruments exhibit pleiotropic effects on the outcome, although it has lower precision and statistical power compared to MR-PRESSO. MR-PRESSO identifies and mitigates the impact of horizontal pleiotropy through outlier detection and removal. If horizontal pleiotropy was detected among selected SNPs, analyses were repeated after excluding these SNPs (Bowden et al., 2015). Finally, we conducted a leave-one-out sensitivity test to evaluate result robustness. This involved sequentially removing individual SNPs and recalculating MR scores to identify any significant deviations from the overall results, thereby ensuring the reliability of our MR analyses (Hemani et al., 2017).

### Reverse MR analysis

We performed reverse MR analysis to explore the causal association between gynecologic tumors and significant bacterial genera. SNPs associated with these tumors served as IVs, while UFs, ECs, OCs, and CCs were considered exposures, and bacterial genera were the outcomes.

All statistical analyses were executed using R 4.0.3 software. We employed the “*TwoSampleMR*” package for IVW, WME, and MR-Egger regression methods, and the “*MRPRESSO*” package for MR-PRESSO detection.

## Results

### The selection of instrumental variables

**FIGURE 1:**
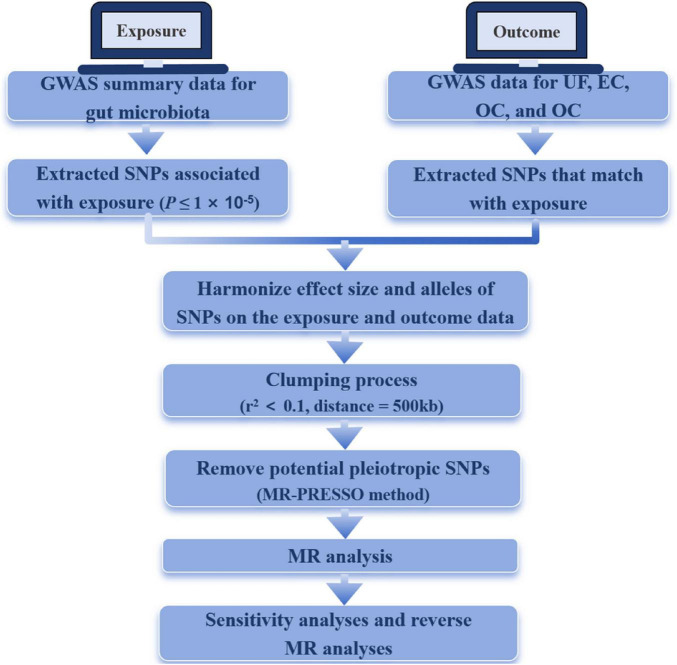
The flowchart of the study. MR, Mendelian randomization; UF, uterine fibroids; EC, endometrial cancer; OC, ovarian cancer; CC, cervical cancer.

Figure 1 shows the study flowchart, and details about the datasets in this study are presented in Table 1. After a series of quality controls steps, we identified the following association: UF was linked to 136 SNPs from 12 bacterial taxonomic groups, EC had 55 SNPs associated with 5 bacterial genera, OC exhibited an association with 65 SNPs from 6 bacterial genera, and CC had an association with 95 SNPs from 8 bacterial genera (Supplementary Table 1). The *F* statistics for the IVs significantly correlated with gut microbiota ranged from 11.701 to 124.169, all exceeding 10. This suggests that the estimates are unlikely to be influenced by weak instrument bias.

**TABLE 1:** Gynecological tumors GWAS samples used in this study.

Study	Trait	Dataset	Case (N)	Control (N)	nSNPs	Ancestry
Discovery	UF	finn-b-CD2_BENIGN_ LEIOMYOMA_UTERI	18060	105519	16379784	European
	EC	ebi-a-GCST006464	12906	108979	9470555	European
	OC	ieu-a-1229	2966	40941	/	European
	CC	ieu-b-4876	563	198523	8506261	European
Replication	UF	bbj-a-157	5954	95010	8877739	East Asian
	EC	ukb-b-13545	1151	461782	9851867	European
	OC	ieu-b-4963	1218	198523	9822229	European
	CC	ukb-b-8777	1889	461044	9851867	European

GWAS, genome-wide association study; nSNPs: number of SNPs; UF, uterine fibroids; EC, endometrial cancer; OC, ovarian cancer; CC, cervical cancer.

### Causal effects of gut microbiota on gynecological tumor

#### UF

At the genus level, the genetic prediction results indicated that the risk of UF was positively associated with an increased relative abundance of *Bacteroides* (OR: 1.178, 95% CI: 1.003–1.383, *P* = 0.046) and *Turicibacter* (OR: 1.129, 95% CI: 1.012–1.259, *P* = 0.029). In contrast, a higher genetically predicted abundance of *Enterorhabdus* (OR: 0.808, 95% CI: 0.688–0.948, *P* = 0.009), *Lachnospiraceae*(OR: 0.882, 95% CI: 0.793–0.982, *P* = 0.022), and *Oscillospira* (OR: 0.874, 95% CI: 0.774–0.988, *P* = 0.031) demonstrated a protective effect against UF. Additionally, there were suggestive causal effects of the phylum *Tenericutes*, class *Mollicutes*, order *Pasteurellales*, and families (*Acidaminococcaceae*, *Bacteroidaceae*, *Bacteroidales S24 7group*, and *Pasteurellaceae*) on UF.

#### EC

The relative abundance of the genus *Butyrivibrio* (OR: 1.083, 95% CI: 1.009–1.163, *P* = 0.022) significantly increased and was positively associated with the risk of EC. Conversely, higher genetically predicted abundances of the genus *Dorea* (OR: 0.796, 95% CI: 0.657–0.964, *P* = 0.020), genus *RuminococcaceaeUCG014* (OR: 0.820, 95% CI: 0.686–0.979, *P* = 0.028), and genus *Turicibacter* (OR: 0.843, 95% CI: 0.735–0.966, *P* = 0.014) were negatively correlated with EC risk. Additionally, we observed that a genetically predicted higher abundance of the family *Acidaminococcaceae* was causally associated with an increased risk of EC (OR: 1.228, 95% CI: 1.018–1.481, *P* = 0.032).

#### OC

At the genus level, the genetic prediction results indicated that increased abundances of *Barnesiella* (OR: 1.395, 95% CI: 1.041–1.869, *P* = 0.026), *Butyrivibrio* (OR: 1.219, 95% CI: 1.048–1.418, *P* = 0.010), and *Lachnospiraceae* (OR: 1.329, 95% CI: 1.019–1.732, *P* = 0.036) were linked to a higher risk of OC. Moreover, higher genetically predicted levels of *Coprobacter* (OR: 0.773, 95% CI: 0.616–0.970, *P* = 0.026) and *RuminococcaceaeUCG010* (OR: 0.644, 95% CI: 0.431–0.962, *P* = 0.032) were associated with a reduced risk of OC. Genetic prediction of a higher abundance of the phylum *Cyanobacteria* was significantly associated with an increased risk of ER^+^ BC (OR: 1.452, 95% CI: 1.132–1.864, *P* = 0.003).

#### CC

**FIGURE 2:**
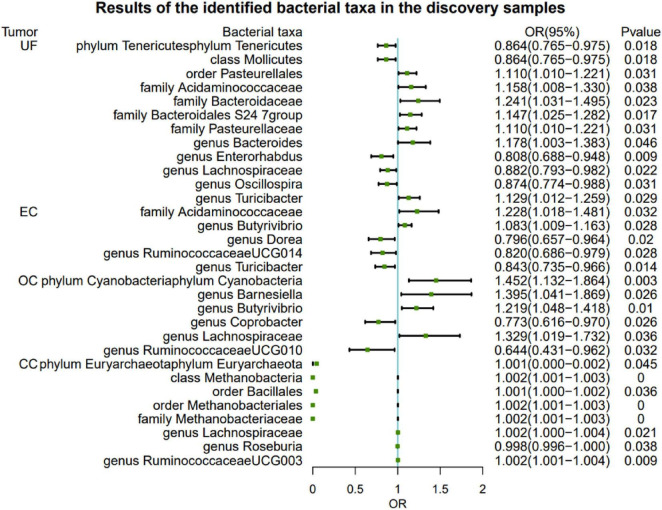
Significant MR analysis results in the discovery samples. MR, Mendelian randomization; SNP, single-nucleotide polymorphism; IVW, inverse-variance weighted; OR, odds ratio; CI, confidence interval; UF, uterine fibroids; EC, endometrial cancer; OC, ovarian cancer; CC, cervical cancer.

The risk of CC was negatively correlated with the levels of genus *Roseburia* (OR: 0.998, 95% CI: 0.996–1.000, *P* = 0.038). However, two other genera, *Lachnospiraceae* (OR: 1.002, 95% CI: 1.000–1.004, *P* = 0.021) and *RuminococcaceaeUCG003* (OR: 1.002, 95% CI: 1.001–1.004, *P* = 0.009), were positively associated with the risk of CC. Furthermore, there were also suggestive causal effects of phylum *Euryarchaeota*, class *Methanobacteria*, orders (*Bacillales* and *Methanobacteriales*) on UF (Figure 2 and Supplementary Table 2).

**FIGURE 3:**
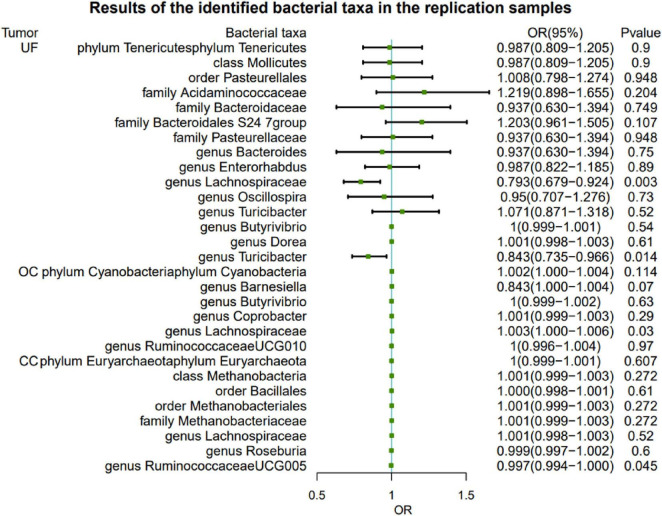
Results of the identified bacterial taxa in the replication samples. MR, Mendelian randomization; SNP, single-nucleotide polymorphism; IVW, inverse-variance weighted; OR, odds ratio; CI, confidence interval; UF, uterine fibroids; EC, endometrial cancer; OC, ovarian cancer; CC, cervical cancer.

To further validate the gut microbiota’s association with gynecologic tumors in the discovery cohort, we conducted additional analysis. As shown in Figure 3, the causal relationship between *Lachnospiraceae* and both UF and OC was consistent with the findings of the discovery cohort, thereby enhancing the credibility of the true causal association (Supplementary Table 3).

### Sensitivity analyses

There is no evidence of heterogeneity among genetic SNPs in *Lachnospiraceae* (Supplementary Table 4). Neither the MR-Egger test nor the MR-PROSSO test provides evidence of horizontal pleiotropy among SNPs (*P* > 0.05, Supplementary Tables 5, 6). Furthermore, the leave-out-analysis demonstrates that the causal association between *Lachnospiraceae* and both UF and OC is not influenced by any single SNP (Supplementary Table 7). The reverse MR analysis shows no evidence of a causal association between UF, OC, and *Lachnospiraceae* (Table 2). Details of the IVs used in the reverse MR analysis are available in Supplementary Table 8.

**TABLE 2:** Reverse causal association between gynecological tumors and gut microbiota.

Exposure	Outcome	nSNPs	OR	95%CI	*P*-value
UF	Lachnospiraceae	125	0.989	0.955–1.025	0.018
OC	Lachnospiraceae	21	0.973	0.934–1.013	0.185

nSNPs is the number of SNPs being used as IVs. MR, Mendelian randomization; SNP, single-nucleotide polymorphism; IVW, inverse-variance weighted; OR, odds ratio; CI, confidence interval; UF, uterine fibroids; CC, cervical cancer.

## Discussion

To investigate the causal relationship between gut microbiota and four common gynecologic tumors (UF, EC, OC, and CC), we performed a two sample MR analysis. By combining the results from both the discovery and validation cohorts, we confirmed a significant association between *Lachnospiraceae* and UF and OC. Specifically, we observed a negative correlation between *Lachnospiraceae* and the risk of UF, as well as a positive correlation with the risk of OC.

The *Phylum Firmicutes* predominates in the gut microbiota of healthy individuals, and *Lachnospiraceae* belongs to this family of anaerobic bacteria. *Lachnospiraceae* has the potential to promote human health by converting primary bile acids into secondary bile acids and producing short-chain fatty acids (SCFAs) such as acetic acid and butyric acid (Sorbara et al., 2020). Research on melanoma patients receiving anti-PD-1 immunotherapy found that a higher abundance of *Lachnospiraceae* was associated with an improved systemic immune response and a positive response to anti-tumor treatment (McCulloch et al., 2022). In colorectal cancer patients, the relative abundance of *Lachnospiraceae* is lower compared to control groups, suggesting that *Lachnospiraceae* may reduce the risk of colorectal cancer and have implications for its prevention and control (Flemer et al., 2018). Studies on gynecological diseases have shown a significantly reduced relative abundance of *Lachnospiraceae* in patients with polycystic ovary syndrome (PCOS) (Wang et al., 2022). Siddiqui et al. (2022) found a decreased quantity of *Lachnospiraceae* in CC patients, while another study demonstrated a positive correlation between *Lachnospiraceae* and persistent HPV infection in CC and a negative correlation with HPV clearance (Ritu et al., 2019). *Lachnospiraceae* is also associated with the risk of BC, as BC patients have a lower relative abundance of *Lachnospiraceae* compared to the control group (Di Modica et al., 2021). However, there is currently limited research on the gut microbiota in relation to UF and OC, and no studies have indicated an association between *Lachnospiraceae* and these gynecological tumors.

Research suggests that dysbiosis of the gut microbiota may be related to an increased risk of gynecological tumors. Specifically, the presence and abundance of *Lachnospiraceae* are associated with estrogen and its metabolites. This bacterial group regulates β-glucuronidase, impacting endogenous estrogen metabolism, leading to the deconjugation and reabsorption of estrogen into the bloodstream, thereby affecting estrogen levels and activity. A significant positive correlation has been observed between *Lachnospiraceae* and circulating estrogen levels (Wu et al., 2023). Elevated estrogen levels in women have been associated with an increased risk of UF, as estrogen promotes the growth of uterine smooth muscle cells, facilitating UF proliferation. On the other hand, the gut microbiota and its metabolites may influence the host’s inflammatory status. Studies have reported an association between UF occurrence and persistent inflammation and immune response (Zannotti et al., 2021). Inflammatory mediators like interleukin (IL)-1, IL-4, and tumor necrosis factor (TNF), as well as immune cells like CD4^+^CD8^+^ T cells, regulatory T cells (Treg, CD4^+^), and follicular helper T cells (Tfh), are significantly elevated in UF patients (K et al., 2023). *Lachnospiraceae* produces SCFAs, which can exert anti-inflammatory and immune-modulatory effects by interacting with the immune system and enhancing intestinal barrier integrity (Ubachs et al., 2021). Our study results indicate that *Lachnospiraceae* might reduce the risk of UF, suggesting a potential protective role in UF development by influencing inflammation and immune response.

As mentioned, *Lachnospiraceae* can influence circulating estrogen levels. The development of OC is also correlated with abnormalities in estrogen synthesis and metabolism. A meta-analysis has shown a higher risk of OC in individuals using hormone replacement therapy (HRT) compared to those who do not use HRT, aligning with our findings, which suggest that *Lachnospiraceae* may increase OC risk by elevating estrogen levels. In addition, the gut-vagina microbiota axis can influence estrogen levels, potentially promoting the onset of estrogen-dependent pathologies such as UF (Takada et al., 2023). Circulating estrogens reach the cells of the vaginal epithelium, stimulating glycogen production, which lactobacilli then metabolize into lactic acid. Typically, Lactobacilli species in the cervicovaginal part of the genital tract play a protective role against OC, which contrasts with our findings. Previous literature has identified members of bacterial, viral, fungal, and parasitic families, suggesting their potential association with cancer initiation and progression (Di Tucci et al., 2023). Parasites infections, such as Trichomonas vaginalis and Schistosoma, as well as viruses like HPV, HIV, and HSV, can alter the microbiota of the female reproductive tract by influencing host immune responses and metabolism. Furthermore, serological studies have linked antibodies against *C.trachomatis*, such as PGp3 and CHSP60-1, with an increased risk of OC. This association may be mediated by the pathogen’s ability to promote survival in DNA-damaged host cells or facilitate the transfer of tubal- derived cells to the growth-promoting microenvironment within the ovaries (Trabert et al., 2019).

However, other studies indicate that SCFAs produced by *Lachnospiraceae* exert anti-tumor effects in OC progression. For instance, butyric acid can inhibit histone deacetylase (HDAC) in OC cells, leading to the transition of tumor cells from the S phase to the G0/G1 and/or G2/M phases, thereby increasing tumor cell apoptosis (Borella et al., 2021). It has been reported that the cervicovaginal microbiome is implicated in OC risk (Sheng et al., 2023). Specifically, cervicovaginal microbes containing less than 50% lactobacilli have been significantly associated with OC (Nené et al., 2019). Additionally, *vaginal Lachnospiraceae*, which produce SCFAs like butyrate by hydrolyzing starch and sugars, show negative correlations with OC development. Despite their potential to promote cancer progression via the induction of regulatory T cells (Tregs), the inhibitory effects of butyrate might outweigh these promotive effects, as butyrate has been shown to interfere with ovarian cancer cell growth *in vitro* (Chen et al., 2021).

While *Lachnospiraceae* may play a role in the development of UF and OC, further research is needed to validate the specific mechanisms in these two types of tumors. This will contribute to a better understanding of the underlying mechanisms in gynecological tumor development and provide more targeted strategies for the prevention, detection, and treatment of UF and OC.

This study has several advantages: Firstly, we conducted a comprehensive investigation of four common gynecological tumors. Secondly, we established causal relationships in the discovery cohort and validated them in an independent validation cohort, enhancing the credibility of the identified causal associations. Thirdly, our MR analysis has identified valuable candidate microbial taxa for subsequent functional research, contributing to the development of novel approaches targeting specific gut microbiota for the prevention and treatment of gynecological tumors.

However, our study has some limitations. Firstly, our research primarily utilized GWAS summary data from European populations, with a small portion of gut microbiota data from other ethnic groups. The variability in data sources may impact the accuracy of our results. Secondly, our bacterial classification was analyzed only at the genus level. Thirdly, the development of gynecological tumors results from multifactorial interactions, with the composition of gut microbiota being influenced by both genetic and environmental factors. Therefore, we cannot exclude the potential influence of interactions between diet and genes, or genes and the environment, on the outcomes. Finally, to further validate the functional role of the identified microbiota, we will conduct *in vitro* experiments to investigate the effects of manipulating the identified microbiota (especially *Lachnospiraceae*) on gynecological tumors such as uterine fibroids and ovarian cancer cell lines. This will include assessing changes in cell proliferation, migration, and apoptosis. Additionally, we plan to conduct *in vivo* studies using animal models to evaluate the therapeutic efficacy of targeting these microbiota.

Our study has established the theoretical foundation for investigating the involvement of gut microbiota in the development and treatment of gynecological tumors, particularly UF and OC. However, further research is required to achieve a deeper understanding of the connection between gut microbiota and gynecological tumors. This includes enlarging the sample size, conducting human cohort studies, and undertaking functional research to provide more precise scientific evidence for the prevention and treatment of gynecological tumors.

## Data availability statement

The original contributions presented in this study are included in this article/Supplementary material, further inquiries can be directed to the corresponding author.

## Ethics statement

Ethical approval was not required for the study involving humans in accordance with the local legislation and institutional requirements. Written informed consent to participate in this study was not required from the participants or the participants’ legal guardians/next of kin in accordance with the national legislation and the institutional requirements. Written informed consent was obtained from the individual(s) for the publication of any potentially identifiable images or data included in this article.

## Author contributions

YX: Writing – original draft, Writing – review and editing. XZ: Conceptualization, Investigation, Writing – review and editing. XN: Software, Writing – review and editing. LZ: Data curation, Writing – review and editing. YS: Validation, Writing – review and editing. AX: Supervision, Writing – original draft.

## Footnotes

1
https://mibiogen.gcc.rug.nl

2
https://gwas.mrcieu.ac.uk/
